# Decreasing Fires in Mediterranean Europe

**DOI:** 10.1371/journal.pone.0150663

**Published:** 2016-03-16

**Authors:** Marco Turco, Joaquín Bedia, Fabrizio Di Liberto, Paolo Fiorucci, Jost von Hardenberg, Nikos Koutsias, Maria-Carmen Llasat, Fotios Xystrakis, Antonello Provenzale

**Affiliations:** 1 Institute of Atmospheric Sciences and Climate (ISAC), National Research Council (CNR), Corso Fiume 4, 10133 Torino, Italy; 2 Meteorology Group, Institute of Physics of Cantabria, CSIC - Universidad de Cantabria, Avda. Los Castros s/n, 39005 Santander, Spain; 3 State Forestry Corps, Viale Antonio Ciamarra 139, 00173 Roma, Italy; 4 CIMA Research Foundation, Via Armando Magliotto 2, 17100 Savona, Italy; 5 Department of Environmental and Natural Resources Management, University of Patras, Seferi 2, 30100 Agrinio, Greece; 6 Department of Astronomy and Meteorology, University of Barcelona, Av. Diagonal 647, 08028 Barcelona, Spain; 7 Institute of Geosciences and Earth Resources (IGG), National Research Council (CNR), Via Moruzzi 1, 56124 Pisa, Italy; Ecole Pratique des Hautes Etudes, FRANCE

## Abstract

Forest fires are a serious environmental hazard in southern Europe. Quantitative assessment of recent trends in fire statistics is important for assessing the possible shifts induced by climate and other environmental/socioeconomic changes in this area. Here we analyse recent fire trends in Portugal, Spain, southern France, Italy and Greece, building on a homogenized fire database integrating official fire statistics provided by several national/EU agencies. During the period 1985-2011, the total annual burned area (BA) displayed a general decreasing trend, with the exception of Portugal, where a heterogeneous signal was found. Considering all countries globally, we found that BA decreased by about 3020 *km*^2^ over the 27-year-long study period (i.e. about -66% of the mean historical value). These results are consistent with those obtained on longer time scales when data were available, also yielding predominantly negative trends in Spain and France (1974-2011) and a mixed trend in Portugal (1980-2011). Similar overall results were found for the annual number of fires (NF), which globally decreased by about 12600 in the study period (about -59%), except for Spain where, excluding the provinces along the Mediterranean coast, an upward trend was found for the longer period. We argue that the negative trends can be explained, at least in part, by an increased effort in fire management and prevention after the big fires of the 1980’s, while positive trends may be related to recent socioeconomic transformations leading to more hazardous landscape configurations, as well as to the observed warming of recent decades. We stress the importance of fire data homogenization prior to analysis, in order to alleviate spurious effects associated with non-stationarities in the data due to temporal variations in fire detection efforts.

## Introduction

Most of the total burned area in Europe occurs in Mediterranean regions, causing severe economic and environmental damage, including the loss of ecosystem services such as carbon sequestration and provisioning of raw materials, the loss of lives and an average of about 4500 *km*^2^ burned every year [1]. In recent decades, changes in climate and other environmental and socioeconomic factors have significantly affected fire regimes [2, 3]. Variations in fire drivers can generate either slow or abrupt modifications in a fire regime that may be difficult to quantify, owing to the limitations in the fire recording protocols and the complexity of the concurrent factors affecting fire activity [4].

Whilst newly available datasets and recent research efforts have significantly boosted our knowledge of forest fires in Mediterranean Europe, analysis of fire trends still shows a rather controversial picture. Many previous studies suggest that fires have increased in Mediterranean regions in recent decades (see, e.g. [2] for a review). However, the limitations of the available fire databases (i.e. the length and homogeneity of the records) could lead to spurious trends and misleading conclusions. In particular, the homogeneity of the fire records could be hampered by the increased efficiency in fire detection and/or recording over time.

Recent studies analyse the multi-decadal fire evolution in Portugal, Spain, France, Italy, and Greece, for instance [5] (1985-2009) and [6] (1985-2005), using the EFFIS Fire database. Nevertheless, an assessment of the influence of data gaps and temporal homogeneity of the data (due to non-stationarities in the fire detection efforts) remains to be made. The same studies focus on the evolution of two standard fire indices: (i) annual burned area (BA) and (ii) annual number of fires (NF), concluding that, generally, BA has decreased and NF has increased. However, the trend estimates for the variable NF, which is more sensitive to the minimum burned area for which a fire is recorded, could be affected by variations in the data recording protocols. On the other hand, most trend studies for the region are limited to smaller spatial domains (see, e.g. [7–10] for Greece, Portugal, Spain and south-eastern France, respectively). Specifically, a significant positive trend in NF and an unclear trend for BA were found analysing the annual fire series for the period 1974-2010 in Greece [7]. In Portugal, analysing the period 1980-2009, no clear trend for the percentage of BA was found, while an unambiguous upwards trend was found for NF [8]. The authors of these studies acknowledge the problem of data homogeneity, noting that the minimum detected burned area has changed over time, and warning of the risk of artefactual trends due to the way data have been gathered. An assessment of the changes of BA and NF in Spain from 1968 to 2010 shows both upward and downward trends for the vegetative season (May-November), and upward trends only in the non-vegetative season, except in the Mediterranean region [9]. Finally, a recent study [10] indicates a general negative trend of fire activity (considering fire season length, fire density and burned area) since the early 1990’s in south-eastern France, a trend which is more pronounced during summer.

The aim of this study is to quantify the trends in burned area and number of fires from long-term regional fire datasets of the European Mediterranean region. Prior to the trend analysis, we undertake a thorough assessment of the fire data at hand (with a focus on their homogeneity), and perform an inter-comparison of several datasets and two different statistical methods for trend significance estimation. In particular, we used for the first time a collection of homogenized databases from various sources, providing an assessment of the uncertainties/deviations associated with data limitations. Here we extend previous studies on fire trends by (i) integrating different available fire datasets in European Mediterranean regions (Portugal, Spain, southern France, Italy, and Greece), (ii) extending the time frame of the analysis at least to the common period 1985–2011, and to even longer time periods depending on the data availability for each particular country, and (iii) performing the trend analysis of BA and NF based on truly homogeneous fire records. To this end, we first consider the fire database of the European Forest Fire Information System (EFFIS; [11]), which provides the official fire statistics of the European Union. We then analyse several databases of different spatial/temporal resolutions from various regional sources (e.g. [7, 8]), with special attention to data integrity and homogeneity.

## Materials and Methods

### Fire data

The European Forest Fire Information System (EFFIS [11]), established by the Joint Research Centre and the Directorate General for the Environment of the European Commission, is the main source of harmonized data on forest fires in Europe. From EFFIS we obtained monthly BA and NF data at NUTS3 level (Nomenclature of Units for Territorial Statistics, which corresponds to aggregations of municipalities or provinces) for Portugal (1980-2011), Spain, southern France and Italy (1985-2011), and Greece (1983-2011). The other regional datasets were obtained from different sources (Table 1):

Portugal the PRFD (Portuguese Rural Fire Database), a publicly available fire database for Portugal, encompassing the period 1980-2005, and thoroughly checked by [8].
Spain EGIF (*Estadística General de Incendios Forestales*), a dataset supplied by the Spanish Ministry of Agriculture, Food and Environment, for the period 1968–2011. Unfortunately, since the data from 1968 to 1973 have several missing values and lack an adequate inter-province homogeneity in the data recording protocols, this period was excluded from the analysis.
France (Mediterranean) PRMT (Prométhée), the official database for forest fires in the French Mediterranean area and operational since 1973, with continuous data from 1974. EGIF and PRMT are among the oldest fire databases in Europe.
Italy the State Forestry Corps (CFS, Corpo Forestale dello Stato) the official fire agency of Italy, which provides fire statistics at the national level, although some autonomous regions (e.g.,Sardinia) keep independent records. CFS provided the fire data for the period 1990-2011, except in Sardinia, where the available period is 1980-2011.
Greece in 1998, the responsibility for recording forest fires was transferred from the Forest Service to the Fire Brigade. From then until 2003, the Forest Service continued to record only the larger fire events and provide forest fire data to EFFIS; yet these series should be considered undervalued. This explains the notation “Please note that the GR data are incomplete, in particular since 1998, when only around 50% of fires are reported” in the data provided by EFFIS and also the fact that data from Greece are marked as “provisional” in EFFIS annual reports. This inconsistency is often raised in scientific publications (e.g. [7] – see the section on wildfire data), and after personal communications with Greek authorities, we concluded that the combination of the two time series, before 1998 (Forest Service) and after (Fire Brigade), results in the best possible fire-statistics data set for Greece (GRC hereafter) although there is a lack of data for the years 1998 and 1999. Some drawbacks arising from the different data sources are addressed accordingly in the results and discussion sections.

**Table 1:** Description of the datasets used in this study. The asterisks indicate the datasets analysed in deeper detail in this study.

Country	Dataset	Period	Reference
Portugal	EFFIS*	1980-2011	[11]
	PRFD	1980-2005	[8]
Spain	EFFIS	1985-2011	[11]
	EGIF*	1974-2011	www.magrama.gob.es
France	EFFIS	1985-2011	[11]
	PRMT*	1974-2011	www.promethee.com
Italy	EFFIS*	1985-2011	[11]
	CFS	1990-2011	www.corpoforestale.it
Greece	EFFIS	1983-2011	[11]
	GRC- Forest service*	1983-1997	[7]
	GRC- Fire brigade*	2000-2009	[7]

The national datasets, as well as the EFFIS dataset, consist of BA and NF occurring in forests and other land areas, excluding agricultural or other artificial surfaces (as detailed in [11]). The raw daily data consist of information for each single fire occurring at the municipality level. These data are aggregated on a monthly scale and assigned to the appropriate NUTS3 unit by means of spatial statistics in a GIS environment. The NUTS classification has been modified several times since its implementation in 2003. In the present study, we used the NUTS3 2006 version (see http://ec.europa.eu/eurostat/web/nuts/ for more details). For each country, two different datasets are available. Hereafter, we focus on the datasets highlighted with an asterisk in Table 1, unless otherwise specified. That is, we concentrate on the longest available datasets for Portugal (EFFIS), Spain (EGIF), France (PRMT) and Italy (EFFIS). However, for one region of the latter country, Sardinia, EFFIS has continuous data only for the period 1997-2011. We thus used the CFS data for Sardinia. For Greece, we considered the GRC dataset, *a priori* more reliable in spite of the missing years. We restricted the analysis to fires larger than 1 ha, a safe minimum value which was detected by all datasets over the whole time period considered (see next Section).

### Trend analysis

We estimated the trends in the annual burned area (BA) and number of fires (NF) on various scales, from the entire domain to national and NUTS3 levels. We express trend estimates as percentages of the change in the 27-year period (1985-2011) with respect to the mean (defined over the whole study period): a BA trend of -50% means that BA decreased by half of its average value.

The linear trends were estimated using the Theil–Sen estimator [12], a non-parametric method that defines the slope as the median slope among all possible lines through pairs of points. Such an approach is less sensitive to outliers than the classic least-squares estimators and is more accurate for non-normal data.

The trend significance was estimated using a bootstrap test [13]. This provides a rigorous and simply interpretable methodology for analysing trends and allows to analyse non-Gaussian distributions. In essence, the method first decomposes the fire series into a linear trend and a time series of residuals, *F*(*t*_*i*_) = *A* + *Bt*_*i*_ + *R*(*t*_*i*_) where *F*, *A* + *Bt*_*i*_ and *R* are, respectively, the fire data (BA or NF), the corresponding linear trend and residuals, and *t*_*i*_ is time. The residuals are re-sampled *N* times (here, *N* = 1000), generating an ensemble of *N* surrogate residual time series, *R*_*s*_(*t*_*i*_), *s* = 1, …, *N*. Each of these is then added to the detected trend, obtaining *N* surrogate fire time series, *F*_*s*_(*t*_*i*_) = *A* + *Bt*_*i*_ + *R*_*s*_(*t*_*i*_). From each of these, a new linear trend estimate *B*_*s*_ is obtained with the Theil–Sen estimator, eventually providing a distribution *P*(*B*_*s*_) of *N* trend estimates from the surrogate data ensemble. The probability of obtaining a zero trend estimate (i.e. whether and how *P*(*B*_*s*_) includes *B*_*s*_ = 0) can then be assessed and, consequently, the significance of the trend estimate *B* from the original data is obtained. For more details on this method, readers are referred to these studies [13, 14]. The code implementing it is publicly available through this link: http://www.am.ub.es/~mturco/codes/testTrend_MT.zip.

We obtained similar results applying the standard Mann-Kendall trend test (see Figure A in S1 File).

## Results

### Database assessment

**Fig 1:**
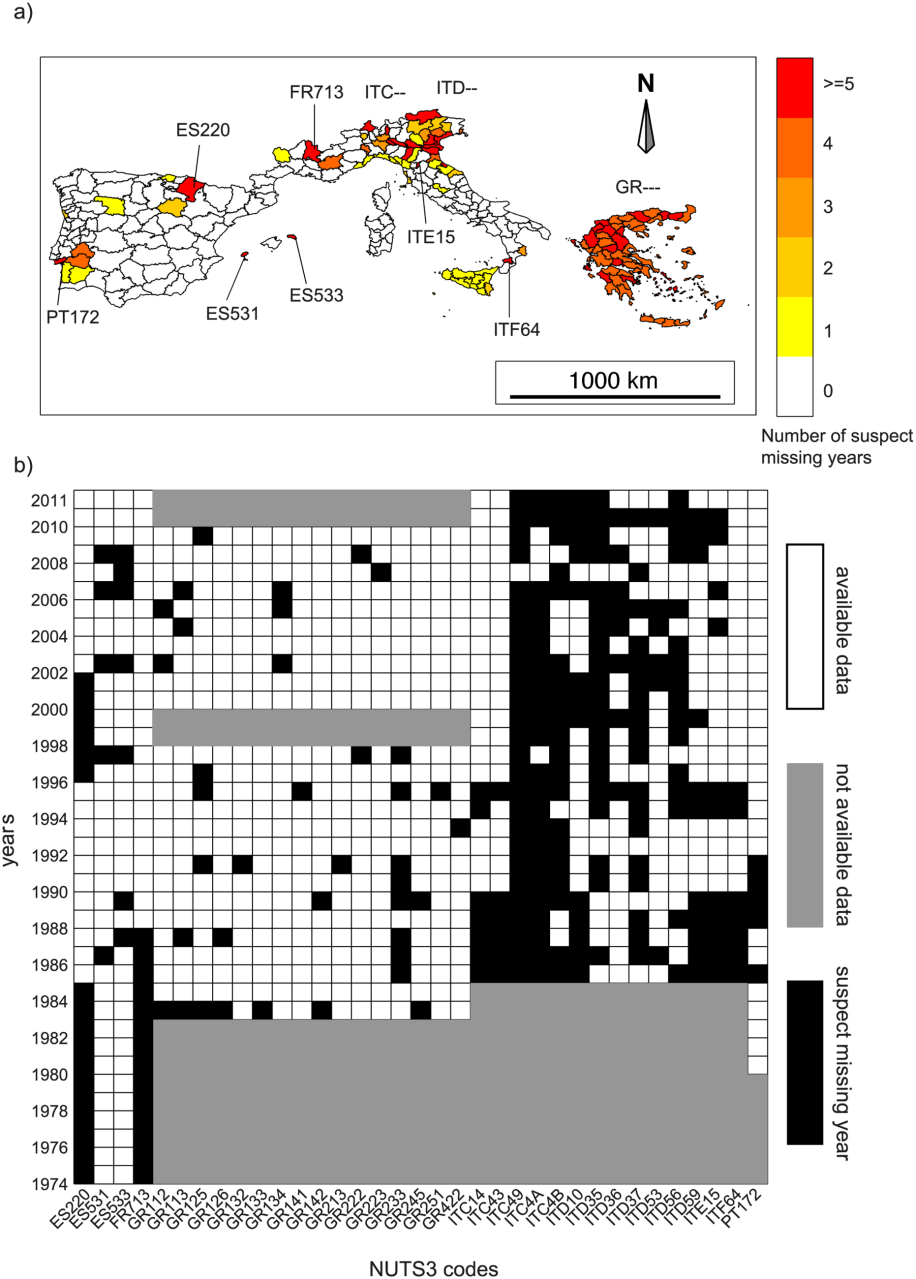
(a) Number of full years with potentially missing data shown over the NUTS3 units map; (b) available data in each year and for each NUTS3 unit where there are at least 5 potentially missing years.

All available time series were checked to ensure that periods with reported burned area and number of fires equal to zero were not mistaken for periods with missing data. For instance, there are some NUTS3 units with no fires for the entire year. This is more likely to happen because there are missing data rather than because there are no fires. To estimate how data series are affected by missing data, we calculated the total burned area for each year and for each NUTS3 unit, and mapped the number of potentially missing years (i.e. total annual burned area in an entire year is equal to zero, Fig 1a). This allowed the identification of “suspicious” areas most likely affected by missing data in the fire records: locally in Portugal (especially in the Setúbal Peninsula, PT172), Spain (in particular in the province of Navarra, ES220), several regions in Italy (especially in northern regions and locally in the Calabria region, ITF64), and most provinces in Greece. The “suspected missing year” for regions with at least 5 years of potentially missing data (35 out of 251 units) are shown in Fig 1b. For example, Navarra province (north-eastern Spain) probably has missing values during the periods 1974-1985 and 1996-2001. As a conservative choice, we excluded all “suspected missing years” from the analysis of their respective regions.

**Fig 2:**
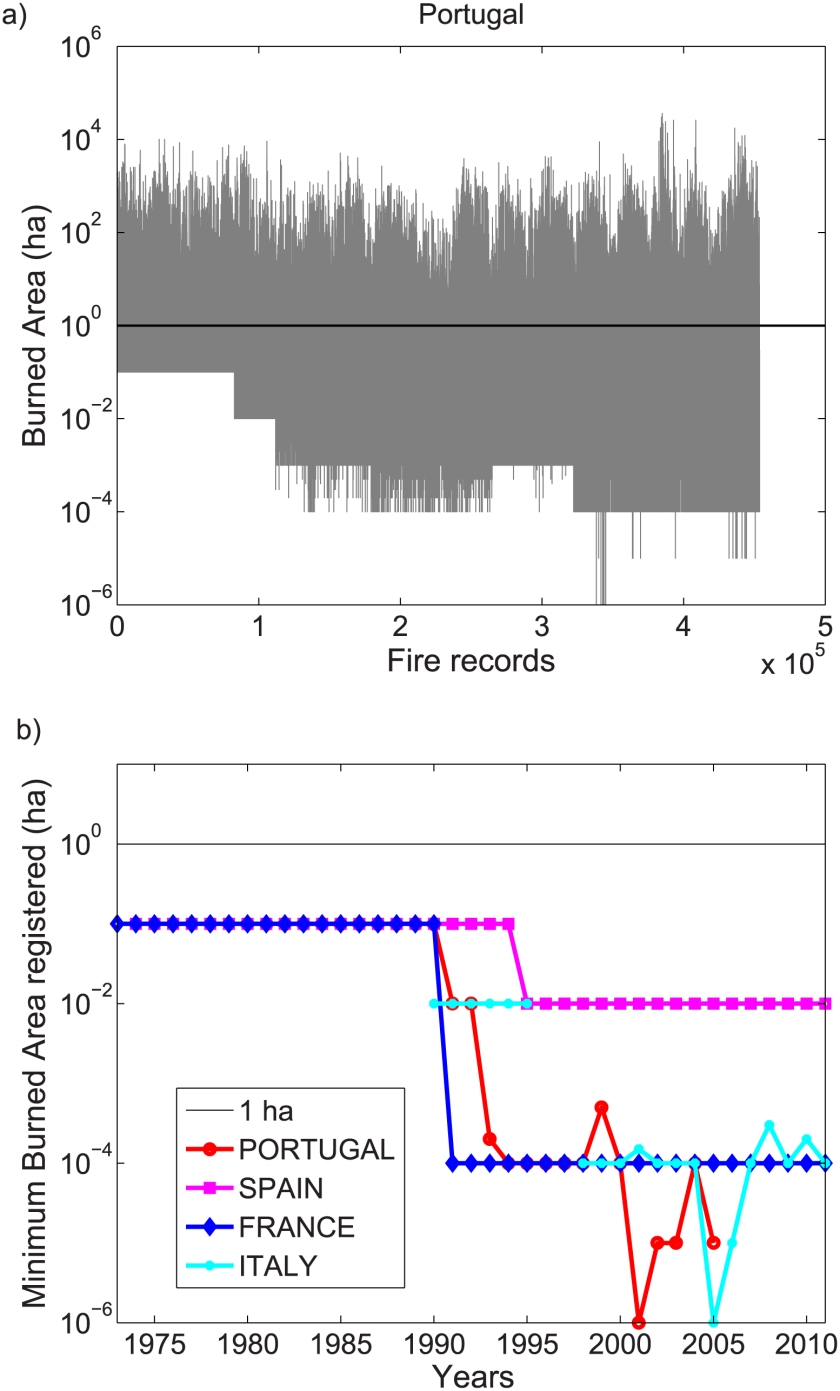
(a) Daily data on burned area (BA in log10 scale) of the PRFD database (Portugal) as a function of time; (b) annual minimum burned area (in log10 scale) for the PRFD (Portugal), SPN (Spain), PRMT (France) and CFS (Italy) datasets.

An important factor hampering trend analyses is data homogeneity. In this sense, it is important to assess whether the minimum burned area for which a fire is recorded is constant over the study area and period [15]. As an example, Fig 2a shows the daily time series of the logarithm of the burned area records in the PRFD database. The plot indicates that the minimum burned area recorded in the data changes over time. Fig 2b shows the annual minimum value for all the countries where raw daily data were available. A common feature is that the minimum area threshold was 0.1 ha until the 1990’s, and then gradually decreased. Currently, even very small fire outbreaks (e.g. burned area < 10^−4^ ha, see Fig 2a) are recorded.

### Trend estimates

**Fig 3:**
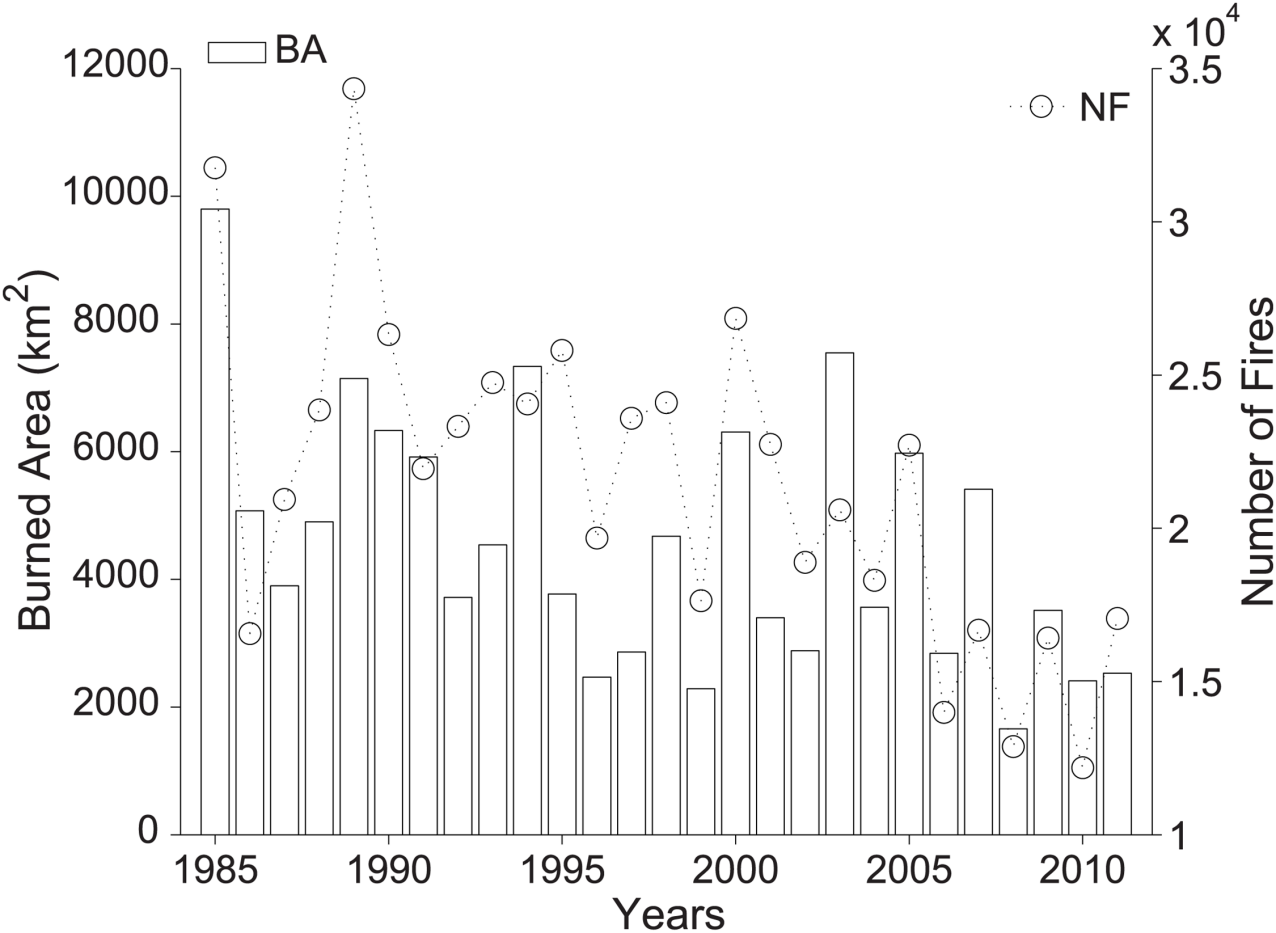
Annual series of the total annual burned area (BA) and number of fires (NF) in Mediterranean Europe.

The magnitude of the estimated trends was often very large. Considering all countries together (Fig 3), the decrease over the 27-year period is -66% for BA (i.e. a decrease of 3020 *km*^2^) and -59% for NF (i.e. a decrease of 12600 fires). These strong negative trends are also confirmed when all units with at least one year of potentially missing data—70 units out of 251—and the periods 1998-1999 and 2010-2011 (unavailable data in Greece, Fig 1b are excluded. In this case, the trends are -68% for BA (2990 *km*^2^) and -57% for NF (11600 fires).

**Fig 4:**
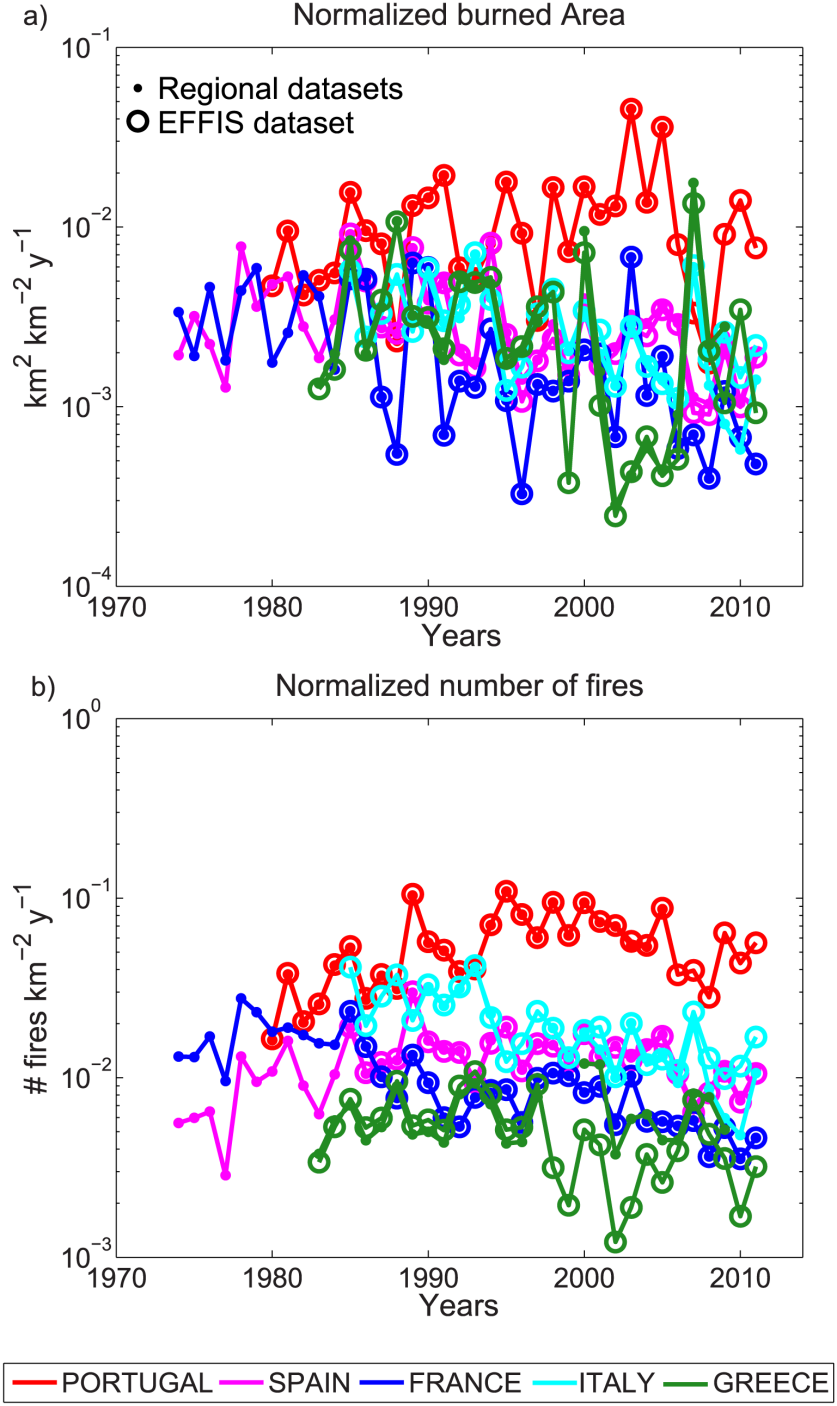
Annual series of (a) the total burned area and (b) number of fires in Portugal, Spain, southern France, Italy and Greece. Both variables are normalized by the area of the corresponding country. The ordinates are on a logarithmic scale (log10).

Fig 4 shows the regional series of BA and NF, normalized by the corresponding study area (in *km*^2^), for Portugal, Spain, southern France, Italy, and Greece. A visual inspection of Fig 4a suggests a non-linear trend, with apparent shifts occurring in the mid-late 1980’s after a nearly negative monotonic BA trend, is visible for all series except that of Portugal. For this country, a positive trend until 2005 is shown. The NF series (Fig 4b) display an overall negative trend in France and Italy. The Spanish and Portuguese series show a positive trend until the late 1980’s—early 1990’s, followed by a negative trend till 2011. The results are more ambiguous in Greece, with an almost steady signal when considering the regional database (GRC), and a negative trend when considering the EFFIS series. For all countries, the fire data from EFFIS and the respective national databases overlap in most cases for their common periods. The most important mismatch between both data sources occurs for the NF values in Greece after 1998.

Focusing on the common period (1985-2011), a significant negative trend for annual series was found in all the studied national domains except Portugal, where a more ambiguous signal was found for both BA and NF series, and Greece, for the NF series (Table 2). To assess whether the observed trends correspond to any particular season, we undertook a seasonal trend analysis (Table 2). Both BA and NF showed a significant negative trend over most seasons/countries, with some exceptions. In Portugal, BA and NF increased in winter and spring, while the trends were not statistically significant in summer and autumn. In Spain, the winter and spring trends were not statistically significant for the BA series, while they were positive for NF. The summer and autumn Spanish series indicated statistically significant negative trends. The BA and NF series in France and Italy displayed negative trends in all seasons. Finally, statistically significant and negative trends for Greece were found for the BA series in spring, summer and autumn, while the NF series showed a statistically significant downward trend only in autumn. These results are fairly insensitive to the exclusion of those NUTS3 units with at least one missing year (See Table A in S1 File).

**Table 2:** Trends for total annual BA and NF for the period 1985-2011. Trend units are the percentages of the total trend for the 27-year period (e.g., ha per 27 years) divided by the historical mean calculated over the whole study period (1985-2011).

Country	Variable	Annual	Winter	Spring	Summer	Autumn
Portugal	BA	-4	+131***	+90***	-6	+7
	NF	+8	+140***	+120***	-37	+12
Spain	BA	-86***	+6	-12	-90**	-71**
	NF	-39**	+45*	+45**	-87***	-78***
France	BA	-73**	-115**	-101***	-63*	-52**
	NF	-89***	-104***	-88***	-116***	-78**
Italy	BA	-78***	-96**	-99***	-46*	-143***
	NF	-101***	-104***	-102***	-71***	-128***
Greece	BA	-91**	+9	-60*	-55**	-145***
	NF	+3	+54	+36	+25	-30**

* *p* < 0.10.

** *p* < 0.05.

*** *p* < 0.01.

**Fig 5:**
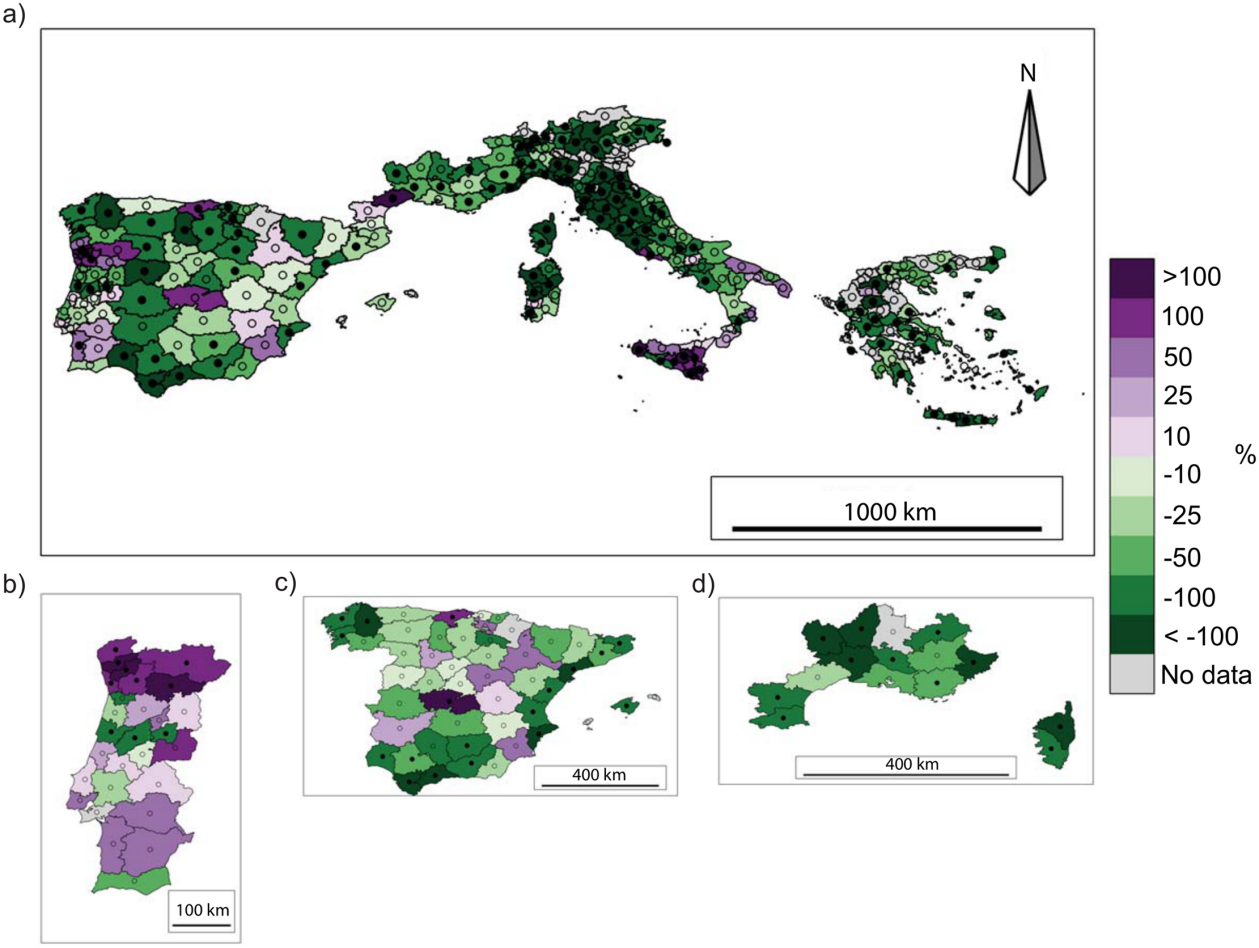
Trends of the annual burned area for (a) the studied domain for the period 1985-2011, (b) Portugal for the period 1980-2011, (c) Spain for the period 1974-2011 and (d) southern France for the period 1974-2011. NUTS3 units with more than 5 years of missing data are excluded from the analysis. Significant trends (*p* < 0.05) are indicated by the filled black circles. Trends are shown as the percentages of the total trend for the available period (e.g. ha per 27 years) divided by the historical mean calculated over the same period (e.g. 1985-2011).

**Fig 6:**
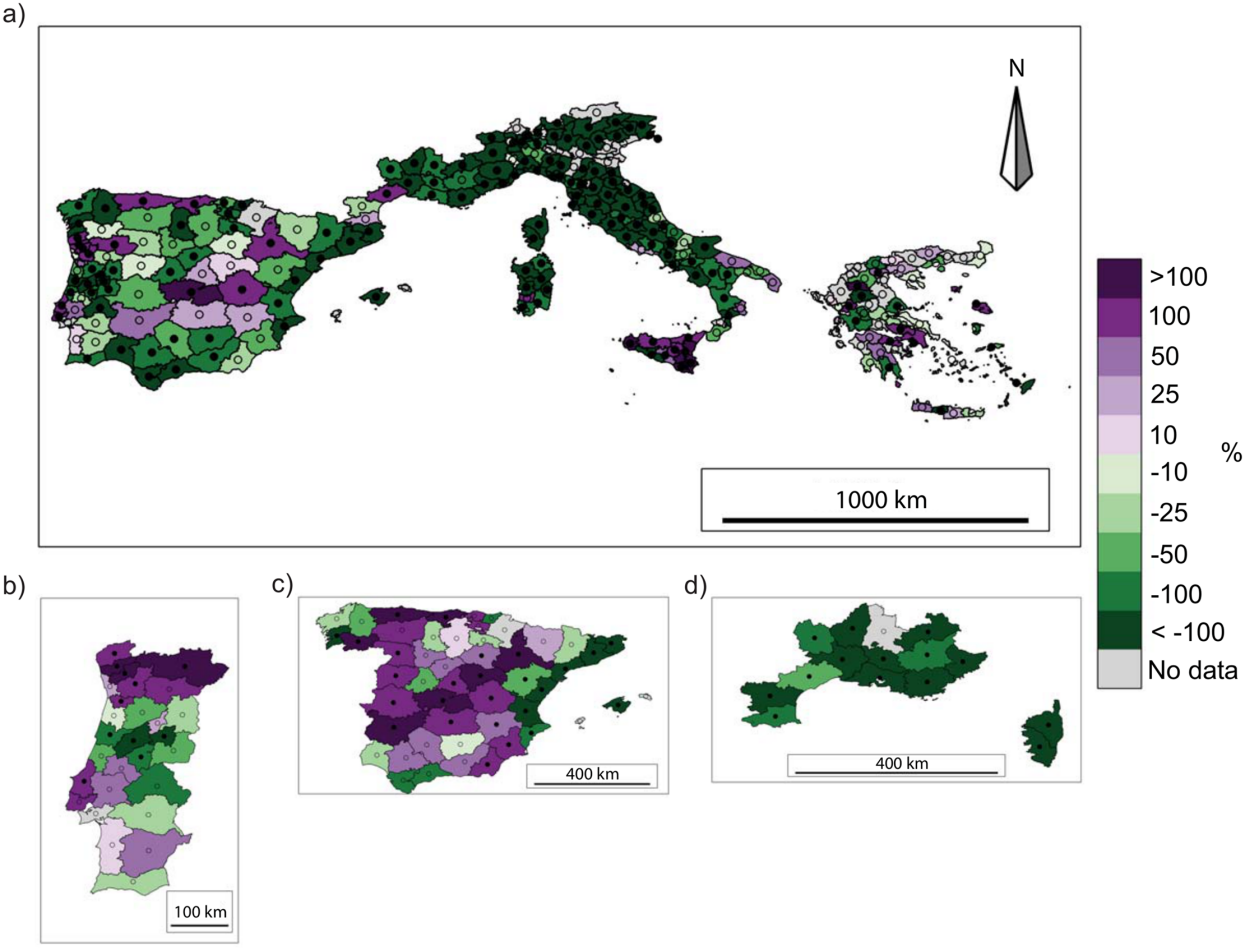
Trends of the annual number of fires for (a) the studied domain for the period 1985-2011, (b) Portugal for the period 1980-2011, (c) Spain for the period 1974-2011 and (d) southern France for the period 1974-2011. NUTS3 units with more than 5 years of missing data are excluded from the analysis. Significant trends (*p* < 0.05) are indicated by the filled black circles. Trends are shown as the percentages of the total trend for the available period (e.g. ha per 27 years) divided by the historical mean calculated over the same period (e.g. 1985-2011).

At NUTS3 level, BA trends over the common period 1985-2011 are quite homogeneous in space (Fig 5a), with a decreasing BA in all domains (not significant in central Spain) except Portugal, where trends are more mixed, and the Cantabria region of northern Spain, Hérault (a department in southern France) and the Italian region of Sicily, where the trends are positive. This pattern generally does not change when a longer period, when available, is considered in the trend analysis (Fig 5b, 5c and 5d). The NF trend shows larger spatial variability, although most of the NUTS3 units have a downward trend. Areas in northern Portugal, central and northern Spain, the French department of Hérault, Sicily, and locally in Greece, however, experienced an increase in NF in the period 1985-2011. Considering longer periods, most provinces in Spain, except the Mediterranean ones, exhibit a positive trend, highlighting non-linearity in trends (Fig 6).

The raw input data of the National and EFFIS datasets are generally similar (the EFFIS database is mainly built from national datasets) so, not surprisingly, the trend estimates are also very similar, with few exceptions (See Figures B, C, D, E and F in S1 File). For Greece, we pointed out strong differences between GRC and EFFIS data that can be attributed to the changes in fire detection procedures (see “Materials and Methods”), leading to a serious problem in fire data consistency.

**Fig 7:**
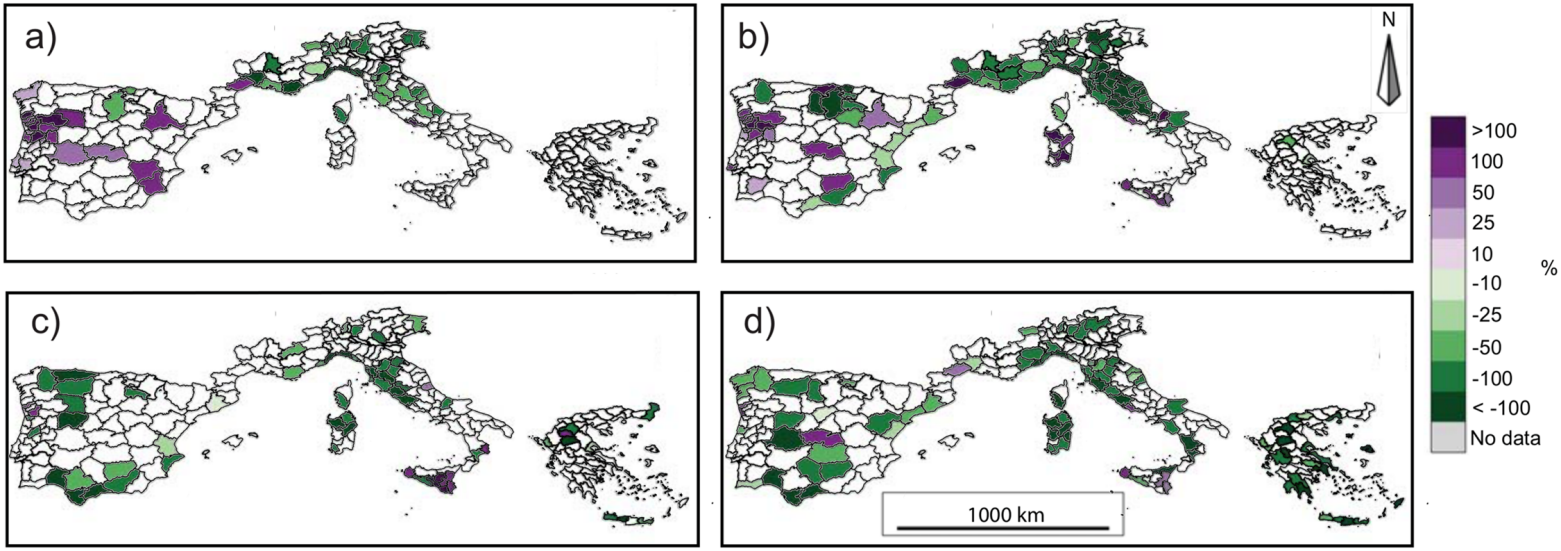
Seasonal trends of the burned area for the period 1985-2011 for (a) December-January-February (DJF), (b) March-April-May (MAM), (c) June-July-August (JJA) and (d) September-October-November (SON). NUTS3 units with more than 5 years of missing data are excluded from the analysis. Only significant trends (*p* < 0.05) are shown. Trends are represented as the percentages of the total trend for the available period (e.g. ha per 27 years) divided by the historical mean calculated over the same period (e.g. 1985-2011).

**Fig 8:**
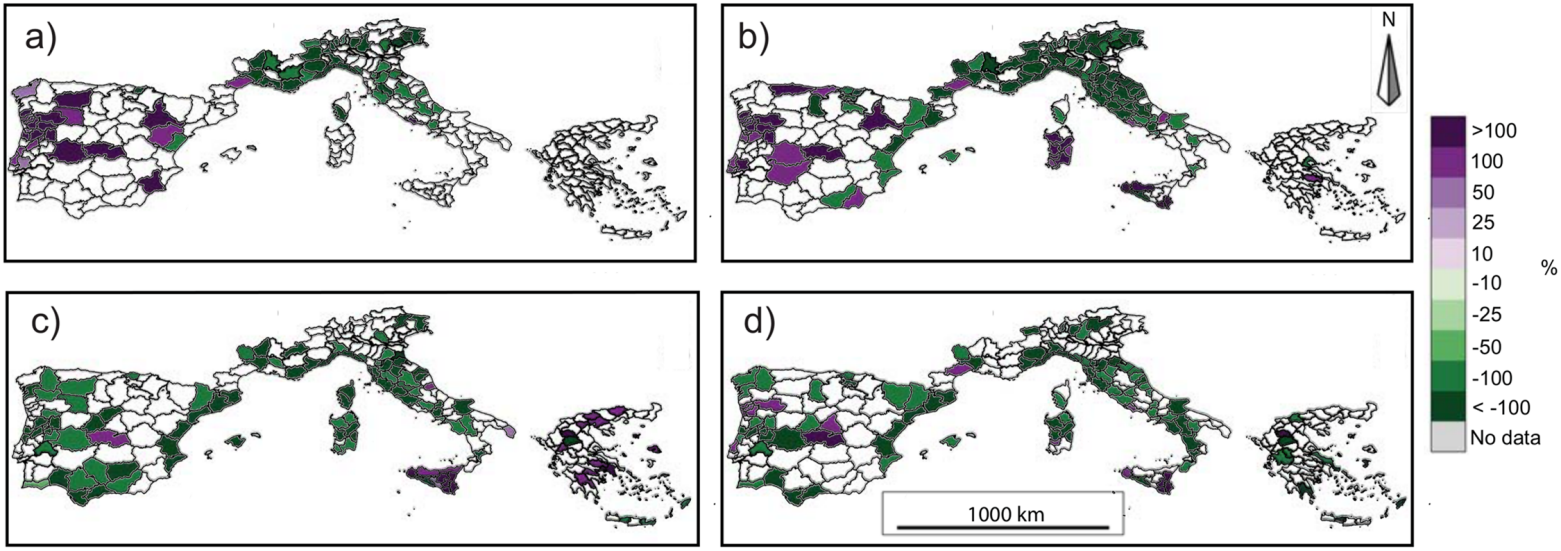
Seasonal trends of the number of fires for the period 1985-2011 for (a) December-January-February (DJF), (b) March-April-May (MAM), (c) June-July-August (JJA) and (d) September-October-November (SON). NUTS3 units with more than 5 years of missing data are excluded from the analysis. Only significant trends (*p* < 0.05) are shown. Trends are represented as the percentages of the total trend for the available period (e.g. ha per 27 years) divided by the historical mean calculated over the same period (e.g. 1985-2011).

In order to better understand which units contribute most to the observed national trends (shown in Table 2), a seasonal analysis at the NUTS3 level was performed (Figs 7 and 8). Generally similar trend patterns emerge with regard to the BA or NF series.

Most of the NUTS3 subdivisions in southern Portugal, Spain, southern Italy and Greece show non-significant trends in the winter series. In contrast, winter BA and NF show a positive trend in northern Portugal and some Spanish provinces, while most of the NUTS3 divisions in southern France and in northern and central Italy exhibit negative trends (Figs 7a and 8a).

Quite similar results are obtained for the spring series, with no significant trends for several regions in southern Portugal, Spain, in the south of peninsular Italy and Greece. Few areas exhibit a significant positive trend: the north of Portugal, the centre of Spain generally, Sardinia, and Sicily. Several Spanish provinces along the Mediterranean coast, southern France, and central and northern Italy show a significant negative trend (Figs 7b and 8b).

The summer BA records show that most of the NUTS3 division have non-significant trends. Several areas in the western and Mediterranean regions of Spain and in central Italy exhibit negative trends, while a few isolated divisions in Portugal, Italy (mostly in Sicily) and Greece show positive trends (Fig 7c). Considering the summer NF series, most of the areas show non-significant or negative trends with rare exceptions: one NUTS3 division in the centre of Spain, Sicily and some regions of Greece (Fig 8c). A similar pattern emerges for autumn, when several NUTS3 divisions exhibit non-significant or negative trends (Figs 7d and 8d).

## Discussion and conclusion

In this work, we present a comprehensive analysis of the trends in fire statistics (burned area and number of fires) for Mediterranean Europe. Special attention was given to avoid sources of error arising from data incompleteness and non-stationarities due to temporal variations in fire detection efforts. To our knowledge, the fire database compiled and analysed here, obtained from a set of different European and national/regional fire data, provides for the first time homogeneous information on fire statistics changes in recent decades in southern Europe. To ensure homogeneity of the time series, we analysed fires with a burned area of at least 1 ha, a safe minimum value which was detected by all datasets over the whole time period considered. Unfortunately, this limitation in the historical data does not make it possible to address the important question of whether the number of small fires has indeed increased or the improvements in fire suppression efforts have led to a decrease in the probability of fires of reaching a burned area of 1 ha.

The agreement of the trend analyses among different databases and reference periods points to a consistent decrease in fire activity in Mediterranean Europe, although with remarkable local exceptions in Portugal and Spain, as discussed afterwards. Overall, even if the trends for individual NUTS3 units must be considered with caution, a general pattern emerges from the analysis of the available data at the southern European level, supporting the validity of the results. Focusing on recent decades (1985–2011), when a common data period is available for Portugal, Spain, southern France and Italy, we show that the annual burned area and annual number of fires have decreased, with the only exception of Portugal, where a mixed signal was found. The results for BA broadly agree with those obtained on longer time scales for which data were available, again yielding predominantly negative trends in Spain and France (1974–2011) and a mixed trend in Portugal (1980–2011). Similar results are found for the number of fires, except for Spain, where an upward trend is found for the longer period (excluding the provinces along the Mediterranean coast) indicating apparent shifts occurring in the mid 1980’s. The seasonal trend analysis indicates that both BA and NF show a significant negative trend over most seasons/regions, with the exceptions of winter and spring in northern Portugal, where significant positive trends in both variables were found, and in some NUTS3 provinces in the interior of Spain.

Even though our findings align with the results of previous studies that indicate an overall decrease in BA [5, 6], they disagree in the NF trends. In contrast to our results, several studies (e.g. [5, 6, 16, 17]) report upward changes in the number of fires, possibly due to differences in the studied period, in the datasets, or the method used to analyse the data. Here, we provide robust trend estimates by addressing data homogeneity in a systematic way, in order to avoid confusing an actual trend with the trend caused by increased efficiency in fire detection. From a scientific standpoint, this work emphasizes the importance of systematically addressing the potential limitations of fire databases, particularly in terms of data homogeneity and the possible gaps that may exist in the records.

Superposed onto these trends, marked year-to-year oscillations are present. Significant correlations exist between the different series when considering the common period 1985-2011. The Spearman’s rank correlation coefficient between the detrended fire series of neighbouring countries is significant (*p* < 0.05): Portugal and Spain (0.52 for BA and 0.75 for NF), Spain and southern France (0.63 for BA), and Italy and Greece (0.69 for BA and 0.72 for NF). This consistent pattern in the interannual variability of fire activity between neighbouring countries may be attributed to meteorological/climatic factors, because certain events driving fires (e.g. droughts, heat waves) usually affect extensive areas beyond national boundaries. In this sense, the sensitivity of burned area to interannual variability of fire-weather conditions has been highlighted for the Mediterranean areas in previous studies (e.g. [18, 19]). This fact also suggests that extreme fire seasons can affect multiple countries simultaneously, justifying the efforts for a transnational network for fire risk management.

Nevertheless, in the forthcoming decades, climate effects could become even stronger and overcome fire prevention efforts, according to the most recent regional [20, 21] and local [22] fire danger projections. This is in keeping with the high sensitivity of the Mediterranean area to the projected climate change in terms of fire activity (e.g. [18, 23, 24]). Yet many uncertainties exist and the direct effect of climate in regulating fuel moisture (e.g. warmer conditions would increase fuel flammability) could be counterbalanced by the indirect effect of climate on fuel load and structure (e.g. warmer conditions might limit fuel availability; [17, 25]), affecting the transition from climate-driven to fuel-limited fire regimes as some regions become drier/warmer [26–28]. The fire database presented here could be used in future works using either statistical [20, 25, 29] or process-based models [30, 31] for a deeper analysis of the fire-climate links, and to estimate the fire response to observed climate trends.

In spite of the climatic controls of fires in these regions, recent trends in fire activity have been shown to be, at least in part, decoupled from climate variability in several Mediterranean countries [32]. In addition to observed climate warming [33, 34], socioeconomic changes, including changes in fuels and landscapes, are relevant factors to explain the observed trends in many areas (e.g. [35]). Indeed, fire regimes result primarily from the interaction of climate, topography, local micro-environments at finer spatial and temporal scales, as well as land use/land cover (LULC) changes [36]. Human intervention may also substantially affect these regimes, while there is extensive debate about the relative importance of weather versus fuel in controlling fire behaviour [37]. It has recently been shown that the synergistic effect between fires and underlying causal factors, such as fuel load and weather conditions, could explain unusually large fire events [38, 39]. Additionally, the effects of fire on ecosystems and landscapes may vary from region to region mainly as a result of local fire history, regeneration patterns and topographic constraints [2, 40], although apart from structural factors, also spatial factors can be considered as causal processes, while their relationships might vary in space [41].

It should be mentioned that multiple and occasionally contradictory types of relationships are found between explanatory and response variables in wildfire occurrence. For instance, demographic shifts from rural to urban areas may favour fuel conditions that lead to large fires, once a fire has been ignited [42, 43]. However, the same population reduction also reduces the probability of (human caused) fire ignition, simply because there are fewer people to start a fire, for whatever reason [41, 44].

For such reasons, there is a potential heterogeneity of fire drivers in space and time that becomes even more evident as the observational scale becomes coarser, as in the case of the NUTS3 level considered in our study. On this scale, we are very likely to observe spatial and temporal heterogeneity of causal factors including the local micro-environment, topography and various socio-economic patterns that obscure the relationship between causes and outcomes and create spatio-temporal variability in wildland fire patterns and trends.

The main anthropogenic drivers controlling fire activity are either direct, via ignition and suppression, or indirect, via fuel management and prevention measures (including changes in population behaviour; [2, 45]). The increased efforts in fire suppression (see, e.g. [46, 47]) have probably played an important role in driving the general downward trends described for most of the Mediterranean area. Indeed, in recent decades fire management strategies have improved, with an increase in fire prevention and fire-fighting measures, new law reinforcements, advances in research and technologies, important efforts in awareness and education, and improved inter-regional collaboration (see, e.g. [1, 9, 48]). We argue that this increased effort in fire management may explain at least part of the observed negative fire trends. One can also argue that the very high fire frequencies reached in recent decades in some Mediterranean regions may reduce the occurrence of new fires and/or their rate of spread when occurring (see [2] and reference therein for a discussion on this issue).

Currently, fire management is focused mainly on fire suppression [49]. However, the situation may become more complex in the future, owing to climate, land-use/land-cover, and ecological and socio-economic vulnerability changes [50, 51]. In addition, fire exclusion and suppression could have a limited long-term positive effect as they can lead to higher fuel load and connectivity [52]. Future fire management strategies should thus expand prevention and adaptation measures, in addition to suppression [51, 53].

Prevention measures should include increasing preparedness especially in the wildland-urban interface, establishing and/or improving early warning systems, for instance setting fire management strategies at a landscape level, promoting the use of fire models and spatial decision support systems (see, e.g. [54, 55]), developing more skilful seasonal predictions able to anticipate potentially dangerous conditions [56], and increasing efforts to reduce ignition probability before and during adverse fire-weather events. In addition, development plans favouring sustainable fuel management should be a key issue to reduce fire risk, combining fuel treatments (mechanical treatments, grazing, prescribed burnings …) and finding the suitable combination for each ecosystem/landscape to increase fuel gaps. In addition, adaptation strategies should incorporate post-fire restoration techniques (e.g. by introducing resprouting woody species and increasing the diversity of species) to increase ecosystem resilience to fire [51, 57]. We conclude by stressing the importance of promoting research activities in relation to all these aspects in order to learn how to coexist with wildfire and improve fire management strategies [58].

## Supporting Information

S1 File Supporting Information. Decreasing Fires in Mediterranean Europe.
(PDF)
