# Supplementary material for: Decreasing Fires in Mediterranean Europe
Source: PLoS One. 2016 Mar 16;11(3):e0150663. doi: 10.1371/journal.pone.0150663 (PMC4794223; doi:10.1371/journal.pone.0150663)
Supplement: S1 File — Decreasing Fires in Mediterranean Europe. (PDF) [file pone.0150663.s001.pdf]

S1 File. Supporting information.
Decreasing Fires in Mediterranean Europe

M. Turco *et al.*

Country	Variable	Annual	Winter	Spring	Summer	Autumn
Portugal	BA	-9	+133***	+94***	-15	+5
	NF	+5	+143***	+127***	-44*	+13
Spain	BA	-86***	+5	-11	-90**	-71***
	NF	-41**	+46*	+47.2**	-89***	-81***
France	BA	-72**	-116**	-107***	-61*	-52**
	NF	-89***	-110***	-78***	-116***	-72**
Italy	BA	-85***	-54*	-100***	-65**	-152***
	NF	-107***	-71***	-96***	-89***	-132***
Greece	BA	-80**	-6	-42*	-53*	-142***
	NF	-4	+38	+30	+26	-47**

SA Table: Trends for country-averaged BA and NF for the period 1985-2011. NUTS3 units with more than 1 year of missing data are excluded from the analysis. Trend units are the percentages of the total trend for the 27-year period (e.g., ha per 27 years) divided by the historical mean calculated over the whole study period (1985-2011). * $p < 0.10$, ** $p < 0.05$, *** $p < 0.01$

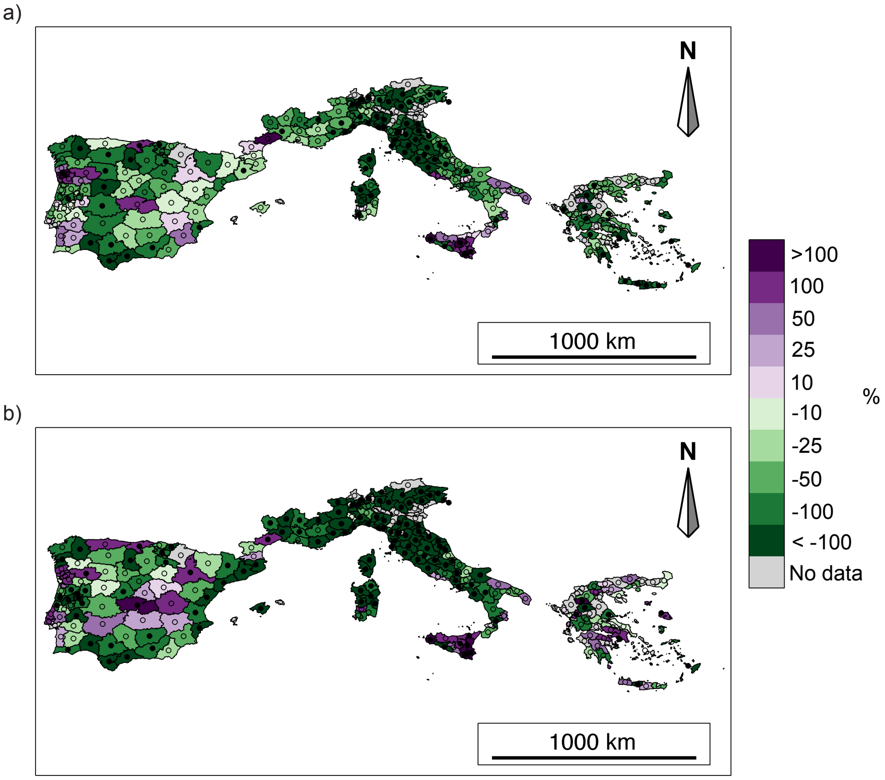

SA Fig: Trends for **(a)** burned area and **(b)** number of fires for the common period 1985-2011. The trend significance is assessed by the Mann-Kendall test. Significant trends are highlighted with filled black points. NUTS3 units with more than 5 years of missing data are excluded from the analysis. Trends are shown as the percentages of the total trend for the available period (e.g., ha per 27 years) divided by the historical mean calculated over the same period (e.g. 1985-2011).

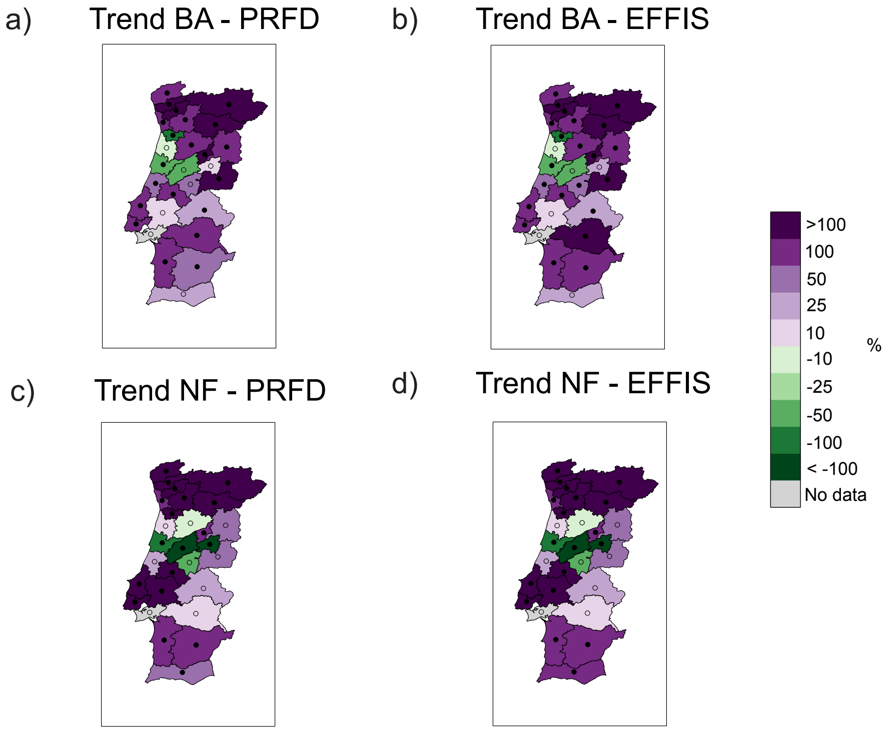

SB Fig: Trend comparison for the period 1980-2005 for Portugal, for **(a)** BA calculated from PRFD data, **(b)** BA calculated from EFFIS data, **(c)** NF calculated from PRFD data and **(d)** NF calculated from EFFIS data. Significant trends are highlighted with filled black points. NUTS3 units with more than 5 years of missing data are excluded from the analysis. Trends are shown as the percentages of the total trend for the available period (e.g., ha per 27 years) divided by the historical mean calculated over the same period (e.g. 1985-2011).

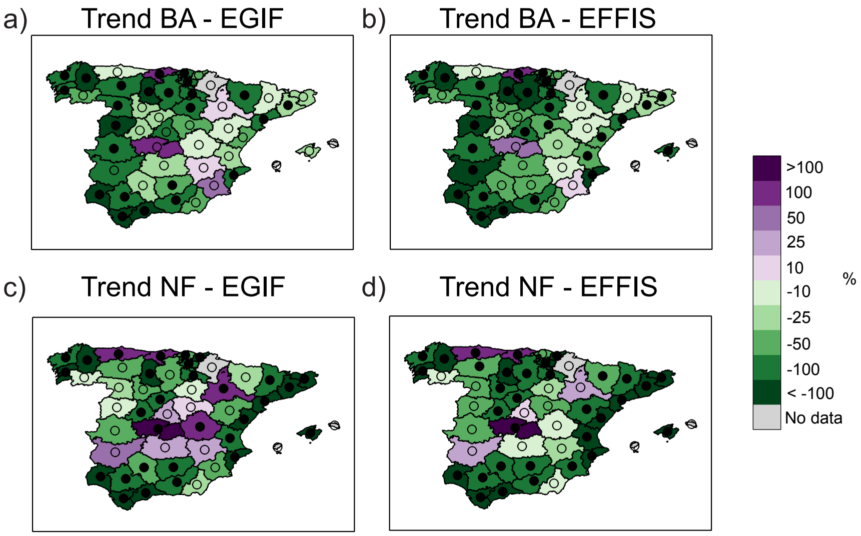

SC Fig: Same as Fig. SB Fig but for Spain. The trends are calculated from EGIF and EFFIS data for the period 1985-2011.

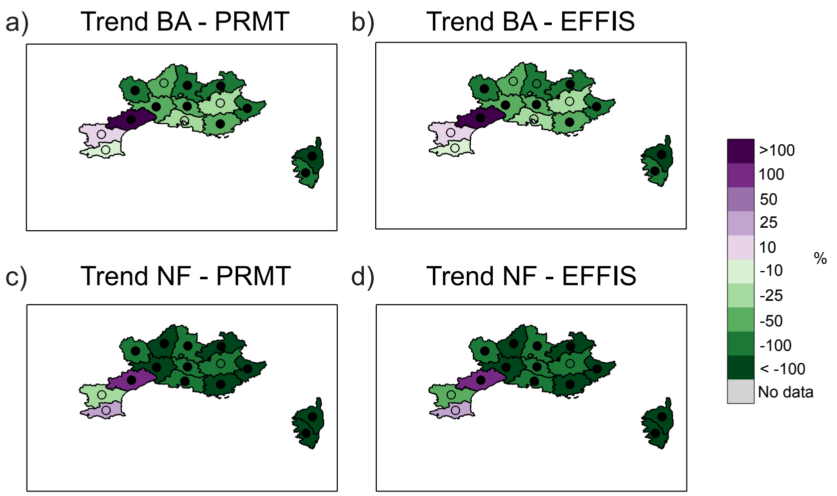

SD Fig: Same as Fig. SB Fig but for southern France. The trends are calculated from PRMT and EFFIS data for the period 1985-2011.

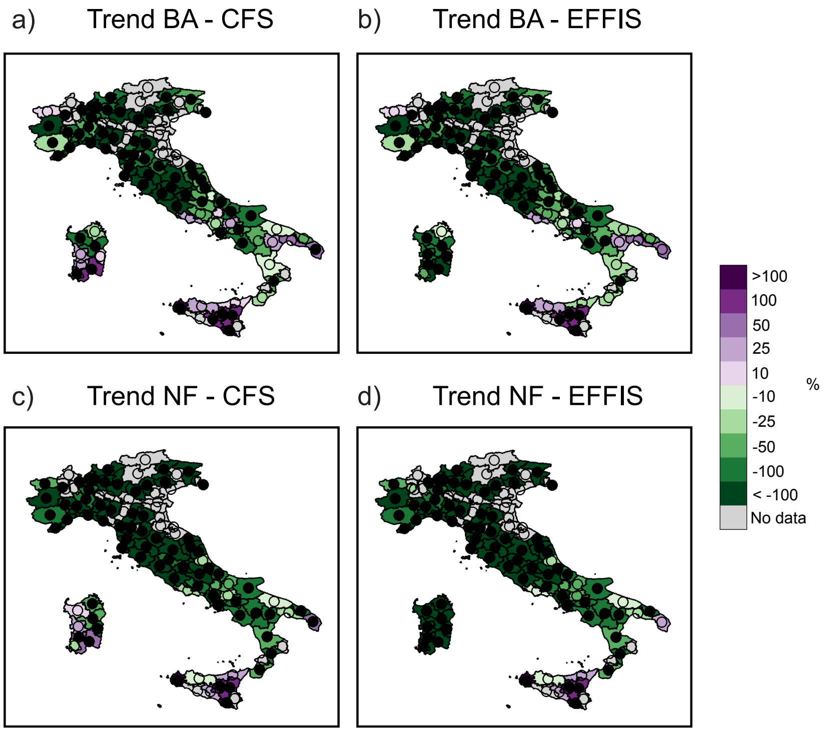

SE Fig: Same as Fig. SB Fig but for Italy. The trends are calculated from CFS and EFFIS data for the period 1990-2011, excluding those areas with less than 15 years of available data (thus allowing to compare the datasets over the Sardinia region; see the paper for more details on data availability).

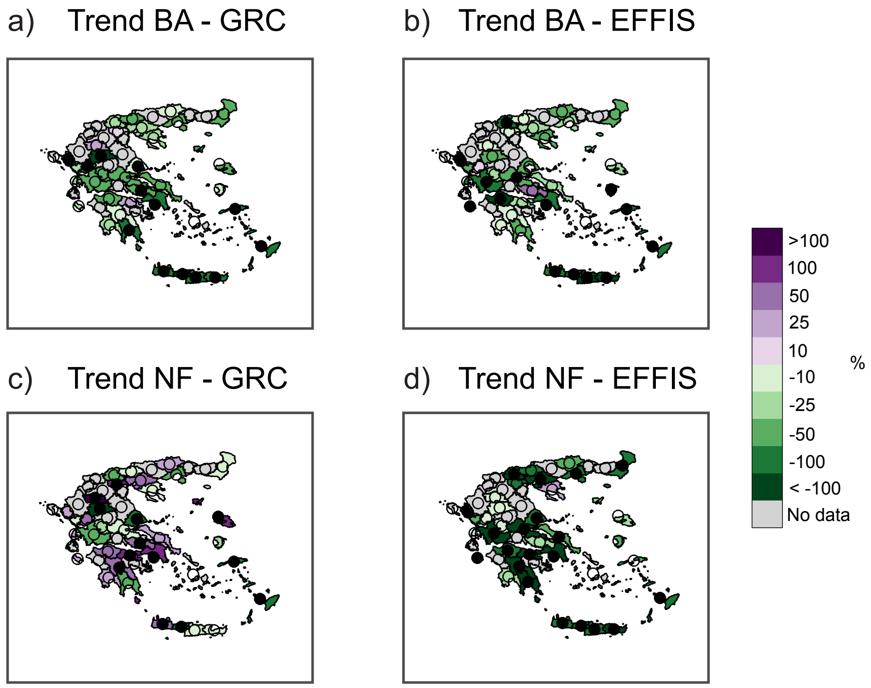

SF Fig: Same as Fig. SB Fig but for Greece. The trends are calculated from GRC and EFFIS data for the period 1983-2009 (excluding the years 1998 and 1999; see the paper for more details on data availability).
